# Comparison of Efficacy and Safety of Taxanes Plus Platinum and Fluorouracil Plus Platinum in the First-Line Treatment of Esophageal Cancer: A Systematic Review and Meta-Analysis

**DOI:** 10.3390/curroncol29090519

**Published:** 2022-09-16

**Authors:** Yue Zhao, Rui Song, Yuanyuan Jia, Xiaoyun Zhang, Shasha Zhang, Chensi Wu, Ruixing Zhang, Zhanjun Guo

**Affiliations:** 1 Department of Gastroenterology and Hepatology, The Fourth Hospital of Hebei Medical University, Shijiazhuang 050011, China; 2 Department of Rheumatology and Immunology, The Fourth Hospital of Hebei Medical University, Shijiazhuang 050011, China

**Keywords:** chemoradiotherapy, esophageal cancer, fluorouracil, prognosis, taxanes, therapeutic effect

## Abstract

Fluoropyrimidine plus platinum (FP) and taxanes plus platinum (TP) are standard treatments for esophageal cancer (EC). This systematic review and meta-analysis aim to explore the difference in the therapeutic effect and toxicity of FP and TP regimens in EC patients. PubMed, Embase, and Cochrane were fully searched and analyzed to find relevant articles on EC patients treated with FP and TP regimens up to 22 March 2022. Thirty-one studies, with a total of 3432 participants, were included in this review. The primary outcomes showed that the prognosis and therapeutic efficacy of TP groups were better than those of FP groups for the EC patients treated with definitive chemoradiotherapy treatment (3-year OS: RR: 1.25, 95% CI: 1.08–1.44, *p* = 0.003; 3-year PFS: RR: 1.43, 95% CI: 1.17–1.75, *p* = 0.0006; ORR: RR: 1.17, 95% CI: 1.06–1.29, *p* = 0.001). However, TP therapy was significantly correlated with a higher incidence of leukopenia and thrombocytopenia (*p* < 0.05). In the preoperative neoadjuvant chemoradiotherapy group, these two groups had a similar survival time (*p* > 0.05). The FP regimen corresponded to a higher incidence of thrombocytopenia, while the TP regimen was associated with an increased incidence of febrile leukopenia (*p* < 0.05). Therefore, TP regimens could generate both superior clinical response and survival benefits when compared with FP regimens in EC patients undergoing definitive chemoradiotherapy.

## 1. Introduction

Esophageal cancer (EC) is one of the most common malignant tumors worldwide [1,2]. GLOBOCAN 2020 reported that EC was listed globally as seventh for incidence and sixth for mortality [2]. Eastern Asia exhibits the highest regional incidence rates, partially because of the large burden in China [3]. The main pathological types of EC are esophageal squamous cell carcinoma (ESCC) and esophageal adenocarcinoma (EAC) [3]. Although the diagnosis and treatment methods have improved significantly in the past decade, the prognosis of EC patients is still lower than expected [1]. At present, surgery still plays a primary role in the treatment of EC; additionally, the therapeutic effect of surgical monotherapy on some locally advanced EC patients is not satisfactory to clinicians [4,5]. Alternatively, neoadjuvant and radical therapies, such as chemotherapy, radiotherapy, or chemoradiotherapy (CRT), have been widely employed to improve the survival rate of EC patients [4,6,7].

Taxanes, platinum, and fluorouracil have long been recognized as important chemotherapeutic drugs in the treatment of EC [8,9]. Taxanes are mitotic inhibitors, which can restrain cell mitosis by promoting tubulin polymerization and inhibiting depolymerization so as to inhibit tumor growth. So far, there has been a wide range of cytotoxic effects on a variety of solid tumors [10]. Tissue culture studies have shown the ability of taxanes to block and/or prolong cells in the G2 or M phase of the cell cycle [11]. It can also induce sensitive cell apoptosis and reduce the consumption of oxygen in solid tumors and, subsequently, increase the local oxygen supply [12]. Fluorouracil antitumor drugs inhibit DNA synthesis by mediating thymine nucleotide synthase activity, which has a beneficial effect on solid tumors, such as gastrointestinal tumors [13]. Fluorouracil can also play a radiosensitizing role by altering the distribution of the cell cycle [14]. At present, CRT is more effective than chemotherapy or radiotherapy alone; it can improve the local control rate, long-term survival rate, and quality of life of EC patients [7,15]. The classical concurrent chemoradiotherapy regimens are those consisting of platinum combined with either fluorouracil or taxanes (paclitaxel or docetaxel) [8,16]. A previous study also indicated that different neoadjuvant chemoradiotherapy (nCRT) or definitive chemoradiotherapy (dCRT) regimens might have dissimilar therapeutic benefits on EC patients [16]. This systematic review and meta-analysis evaluate the difference in the efficacy and toxicity of fluoropyrimidine plus platinum (FP) and taxanes plus platinum (TP) regimens in EC patients undergoing nCRT or dCRT.

## 2. Materials and Methods

### 2.1. Data Sources

This review was performed in accordance with the Preferred Reporting Items for Systematic Reviews and Meta-Analyses (PRISMA) statement [17]. Screening and data extraction processes were conducted by two independent reviewers, and the differences were resolved by a third reviewer. Multiple electronic databases, including Pubmed, Embase, and Cochrane, were systematically searched to find all the available articles published before 22 March 2022. The keywords or MeSH headings referenced were “Esophageal Neoplasms”, “Esophageal Squamous Cell Carcinoma”, “Adenocarcinoma of Esophagus”, “Taxoids”, “Paclitaxel”, “Docetaxel”, “Fluorouracil”, “Capecitabine”, “S1”, and “Chemoradiotherapies”. It should be noted that only human studies in the English language were considered for inclusion. 

### 2.2. Inclusion Criteria

(1) Study design type: randomized controlled trial (RCT), cohort study, or case-control study; (2) Study object: patients diagnosed with EC (ESCC or EAC), as confirmed by pathological evidence; (3) Intervention measures: dCRT or nCRT. The first-line chemotherapy regimens were taxanes (paclitaxel or docetaxel) plus platinum and fluorouracil (5-FU or capecitabine or S1) plus platinum; (4) Study outcome: 3-year survival data, objective response rate (ORR), pathologic complete response (pCR), R0 resection rate, and grade 3 or above toxicity, which could be obtained from the article or survival curve.

### 2.3. Exclusion Criteria

(1) Articles that were published repeatedly; (2) Articles lacking full texts and responses from the corresponding authors; (3) Articles that were reviews, meeting summaries, single-case reports, abstracts, expert consensuses, or editorials; (4) Studies with incomplete raw data; (5) Studies including patients with recurrence or metastasis after surgery and chemoradiotherapy; (6) Studies with too small a sample size (sample size < 30).

### 2.4. Data Extraction and Evidence Evaluation

According to the chemotherapy regimens used, EC patients were divided into two groups: TP and FP. They were further divided into two subgroups, namely, nCRT and dCRT groups, according to the subsequent surgical intervention. The outcomes measured were 3-year overall survival (OS) and 3-year progression-free survival (PFS). The ORR of the dCRT groups and the pCR and R0 resection of the nCRT groups were also included. The following data were extracted from the included articles: the first author, publication year, geographical region, pathological type, chemotherapy regimen, median radiotherapy dose, number of patients, treatment strategy, clinical stage, follow-up time, 3-year OS and PFS, ORR, pCR, R0 resection rate and grade 3 or above toxicity. When the 3-year OS and PFS were not directly provided in the article, they were extracted from the survival curve using Eagauge Digitizer software. The extracted data were sorted using standard tables. The quality of the RCTs was evaluated using the Cochrane risk bias evaluation tool. The evaluation results were divided into high bias risk, low bias risk, and unknown bias risk. Cohort studies or case-control studies were assessed with the Newcastle Ottawa scale. There were 9 stars in the article quality evaluation, and articles with 6 stars or more were retained.

### 2.5. Statistical Methods

RevMan 5.3 analysis software was used to statistically analyze the relevant outcome indicators. The summary measure was the risk ratio (RR) with a 95% confidence interval (95% CI) for 3-year OS and 3-year PFS, and *p* < 0.05 was considered statistically significant. *Q* tests and *I*^2^ tests were introduced to evaluate the heterogeneity of the results. According to relevant standards in the Cochrane Intervention System Evaluation Manual, the fixed-effect method was used if the heterogeneity was acceptable (*I*^2^ < 50%, *p* > 0.10). Once the heterogeneity was established (*I*^2^ ≥ 50% and *p* ≤ 0.10), a random effect model was performed. The sources of heterogeneity, such as methodological heterogeneity, statistical heterogeneity, and clinical heterogeneity, should be analyzed, and sensitivity analysis could be used to exclude a single study. The sensitivity analysis was carried out by Stata software version 14.0, and the risk of publication bias was determined using *Begg’s* tests and *Egger’s* tests. When *p* > 0.05, there was considered to be no publication bias. If the number of included articles was less than 10, no further bias test was required.

## 3. Results

### 3.1. Selection of Studies

**Figure 1:**
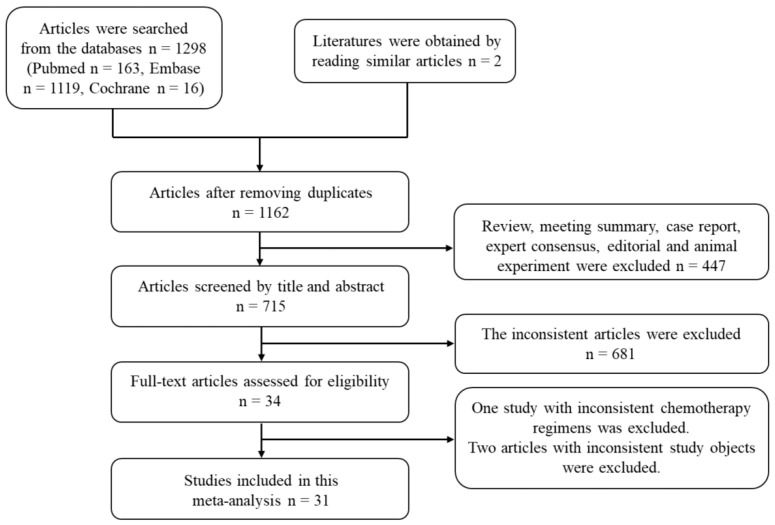
Flow diagram of the selection process for included studies in the systematic review.

The two evaluators developed retrieval strategies, respectively. Through preliminary inspection, a total of 1300 relevant studies were obtained, including 1298 which met the inclusion criteria and 2 similar reading articles. After further screening, 34 articles were obtained for further evaluation [18,19,20,21,22,23,24,25,26,27,28,29,30,31,32,33,34,35,36,37,38,39,40,41,42,43,44,45,46,47,48,49,50,51]. Among these studies, the chemotherapy regimen of one and the study objects of two did not meet the inclusion criteria. Finally, a total of 31 articles involving 3432 participants were included [18,19,20,21,22,23,24,25,26,27,28,29,30,31,32,33,34,35,36,37,38,39,40,41,42,43,44,45,46,47,48]. The detailed retrieval process of this review is shown in Figure 1.

Among these articles, 14 were from China, 4 were from the United States, 3 were from Germany, 2 were from Canada, 2 were from the Netherlands, 1 was from Italy, 1 was from Australia, 1 was from India, 1 was from Thailand, and 2 were multicenter studies. A total of 12 articles included the treatment strategy of dCRT or concurrent chemoradiotherapy (CCRT), 14 involved nCRT, and 5 included dCRT/CCRT and nCRT. Among the 3432 patients included, 2477 had ESCC, 945 had EAC, and 10 had other pathological types. The characteristics of these selected studies are shown in Table 1 and Table S1.

### 3.2. Quality Evaluation

**Figure 2:**
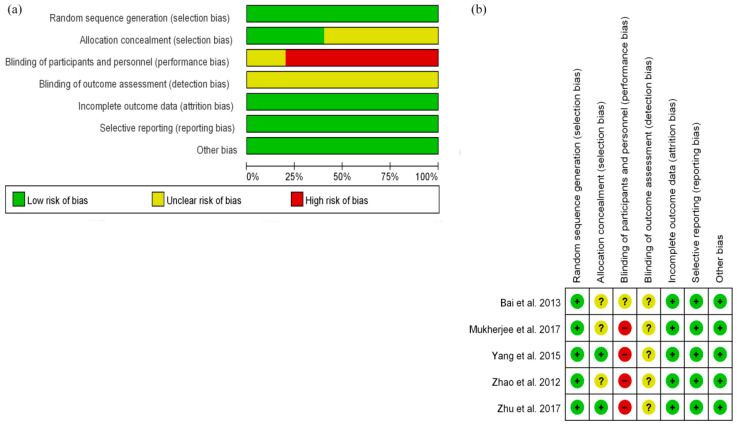
The Cochrane risk bias of five randomized controlled trials. (**a**) risk of bias graph; (**b**) risk of bias summary.

Five RCTs with a low risk of bias evaluated using the Cochrane risk bias were included in this study (Figure 2). The 26 cohort studies had a medium-to-high quality, as assessed by the Newcastle Ottawa scale.

### 3.3. Survival Outcome

#### 3.3.1. TP Results in Better Disease Control and Long-Term Survival Compared with FP in the dCRT Group

**Figure 3:**
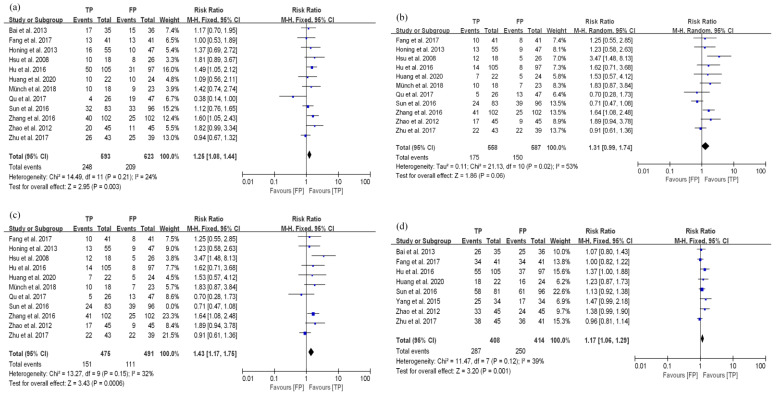
Analyses of curative effects in TP and FP groups in EC patients treated with dCRT. (**a**) 3-year OS; (**b**) 3-year PFS; (**c**) 3-year PFS after correction; (**d**) ORR.

A total of 12 studies reported 3-year OS in EC patients treated with dCRT. In these studies (ESCC vs. EAC: 1118 vs. 98), the pathological types included two mixed ESCC and EAC cases [23,25], and the rest only included ESCC. The summary results illustrated that the 3-year OS of the TP group was better than that of the FP group (RR: 1.25, 95% CI: 1.08–1.44, *p* = 0.003), and there was no heterogeneity in these results (*I*^2^ = 24%, *p* = 0.21, Figure 3a).

The difference in the 3-year PFS of TP and FP regimens in EC patients receiving dCRT was compared across eleven studies. Among these studies (ESCC vs. EAC: 1047 vs. 98), two included ESCC and EAC [23,25], and the remaining studies included ESCC. Due to the heterogeneity of the results (*I*^2^ = 53%, *p* = 0.02, Figure 3b), a sensitivity analysis was conducted on the 11 studies. The study by Sun et al. [24] had a great impact on the heterogeneity, which might have been related to the inclusion of multiple chemotherapy regimens in this study (Figure S1a). After removing this study, there was low heterogeneity in the remaining studies (*I*^2^ = 32%, *p* = 0.15). Compared with the FP group, the TP group had better 3-year PFS results (RR: 1.43, 95% CI: 1.17–1.75, *p* = 0.0006, Figure 3c).

A total of 8 studies reported the ORR in 822 ESCC patients treated with dCRT. We found that the TP group had better ORR results than the FP group (RR: 1.17, 95% CI: 1.06–1.29, *p* = 0.001), which had low heterogeneity (*I*^2^ = 39%, *p* = 0.12, Figure 3d).

#### 3.3.2. TP and FP Regimens Had Similar Survival Efficacy in EC Patients Treated with nCRT

**Figure 4:**
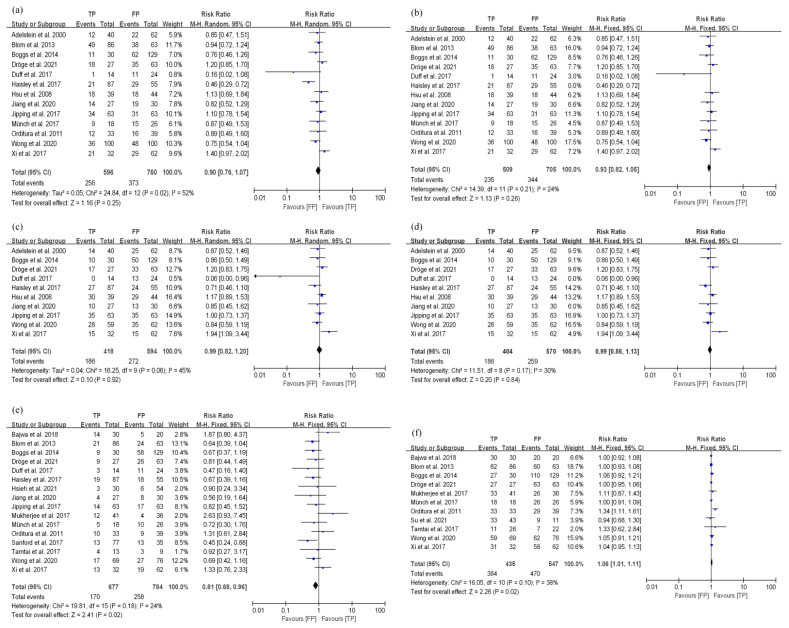
Analysis of curative effects of TP and FP groups in EC patients treated with nCRT. (**a**) 3-year OS; (**b**) 3-year OS after correction; (**c**) 3-year PFS; (**d**) 3-year PFS after correction; (**e**) pCR; (**f**) R0 resection.

For patients that underwent nCRT, the 3-year OS of TP and FP regimens was compared across 13 studies. Among these studies (ESCC vs. EAC: 740 vs. 616), 7 included ESCC and EAC, and the remaining only included ESCC. Due to the high heterogeneity (*I*^2^ = 52%, *p* = 0.02, Figure 4a), a sensitivity analysis was performed (Figure S1b). After excluding the study by Haisley et al. [40], which seriously affected the heterogeneity, the heterogeneity result was acceptable (*I*^2^ = 24%, *p* = 0.21, Figure 4b). Furthermore, no significant difference in the 3-year OS of TP and FP groups (RR: 0.93, 95% CI: 0.82–1.05, *p* = 0.26, Figure 4b) could be found.

This review included 10 studies on the 3-year PFS in EC patients receiving nCRT, among which 4 included ESCC plus EAC, and 6 included ESCC (ESCC vs. EAC: 580 vs. 432). There was high heterogeneity in the summary results (*I*^2^ = 45%, *p* = 0.06, Figure 4c). Through sensitivity analysis, it was found that the study by Duff et al. [41] might have a great impact on the heterogeneity because of its small research data (Figure S1c). The remaining studies were not heterogeneous after removing this study (*I*^2^ = 30%, *p* = 0.17). The results showed that no difference was found in the 3-year PFS of TP and FP groups (RR: 0.99, 95% CI: 0.86–1.13, *p* = 0.84, Figure 4d).

A total of 16 studies reported the pCR in EC patients treated with nCRT. Among these studies (ESCC vs. EAC: 822 vs. 639), 10 included ESCC and EAC cases, 5 reported ESCC cases, and 1 included EAC. The summary result showed that the pCR in the FP group was better than that of the TP group (RR: 0.81, 95% CI: 0.68–0.96, *p* = 0.02). These results had low heterogeneity (*I*^2^ = 24%, *p* = 0.18, Figure 4e).

There were 11 studies comparing the R0 resection rates of TP and FP regimens in EC patients undergoing nCRT. In these studies (ESCC vs. EAC: 719 vs. 263), the pathological types included six mixed ESCC and EAC types, four only included ESCC, and one included EAC. The summary results showed that the R0 resection rate of TP was better than that of the FP group (RR: 1.06, 95% CI: 1.01–1.11, *p* = 0.02), and there was no heterogeneity in this result (*I*^2^ = 38%, *p* = 0.10, Figure 4f).

#### 3.3.3. Bias Test

**Figure 5:**
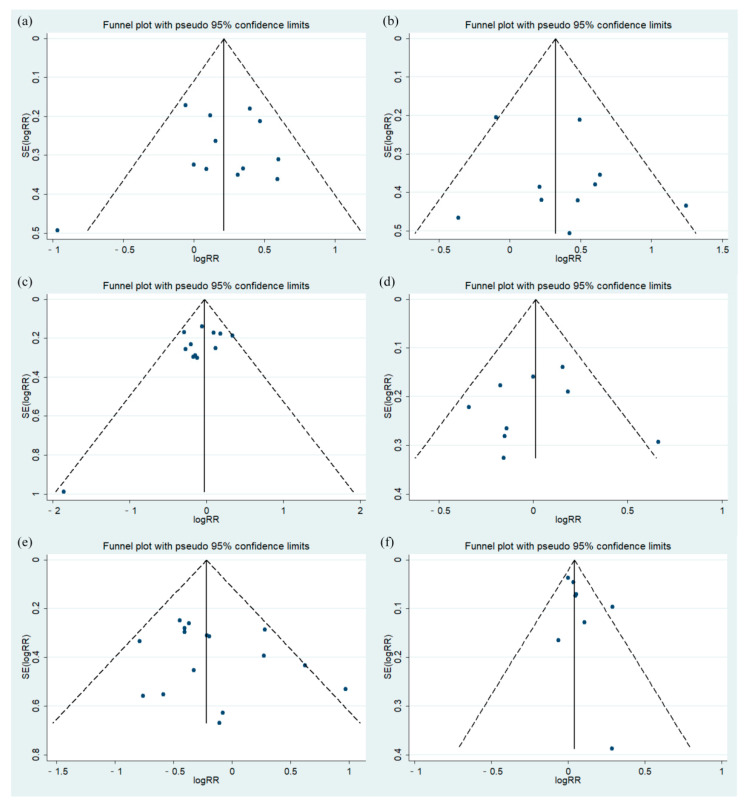
Funnel plots for publication bias. (**a**) 3-year OS in dCRT; (**b**) 3-year PFS in dCRT; (**c**) 3-year OS in nCRT; (**d**) 3-year PFS in nCRT; (**e**) pCR; (**f**) R0 resection.

The outcome indices for the 3-year OS, 3-year PFS, pCR, and R0 resection rate in dCRT and nCRT groups were analyzed (Figure 5). The *p* values of *Begg’s* tests and *Egger’s* tests were greater than 0.05, which suggested that there was no publication bias in this study (Table 2).

### 3.4. Toxicity

**Figure 6:**
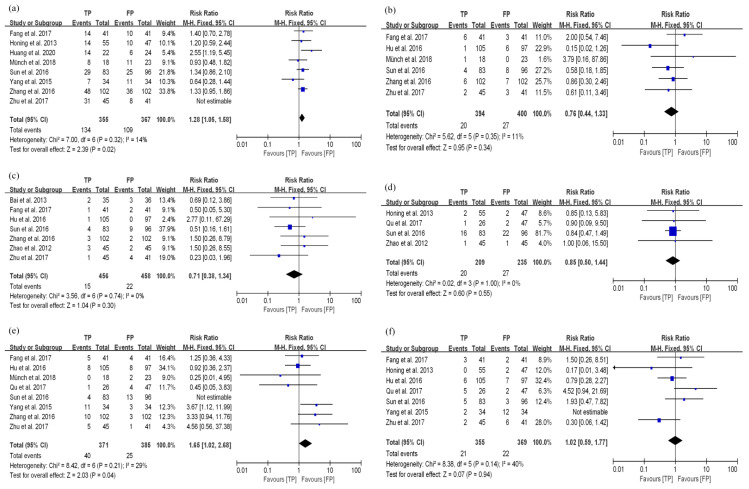
The related toxicity of TP and FP in EC patients treated with dCRT. (**a**) leucopenia; (**b**) anemia; (**c**) pneumonia; (**d**) mucositis; (**e**) thrombocytopenia; (**f**) nausea/vomiting.

Within this study, the related toxicity (grade ≥ 3) of TP and FP regimens in EC patients treated with dCRT was summarized. Patients receiving TP regimens tended to have a higher incidence of leucopenia than those undergoing FP regimens (RR: 1.28, 95% CI: 1.05–1.58, *p* = 0.02, Figure 6a). There were no significant differences in the incidence of anemia (RR: 0.76, 95% CI: 0.44–1.33, *p* = 0.34, Figure 6b), pneumonia (RR: 0.71, 95% CI: 0.38–1.34, *p* = 0.30, Figure 6c), and mucositis (RR: 0.85, 95% CI: 0.50–1.44, *p* = 0.55, Figure 6d) between TP and FP groups. The above results had low heterogeneity (*I*^2^ < 50%, *p* > 0.10). A total of eight articles on thrombocytopenia caused by chemotherapy regimens were included. The results illustrated a certain heterogeneity (*I*^2^ = 52%, *p* = 0.04). Further sensitivity analysis also established that the study by Sun et al. [24] had a great impact on the heterogeneity due to its inclusion of multiple chemotherapy regimens (Figure S1d). After removing this study, there was no heterogeneity in the remaining studies (*I*^2^ = 29%, *p* = 0.21), and the TP group had a higher incidence of thrombocytopenia when compared with the FP group (RR: 1.65, 95% CI: 1.02–2.68, *p* = 0.04, Figure 6e). A total of seven articles on nausea/vomiting caused by these two chemotherapy regimens were included. After correcting the heterogeneity through sensitivity analysis (Figure S1e), the results showed that there was no significant difference in the incidence of nausea/vomiting between the two groups (RR: 1.02, 95% CI: 0.59–1.77, *p* = 0.94, Figure 6f).

**Figure 7:**
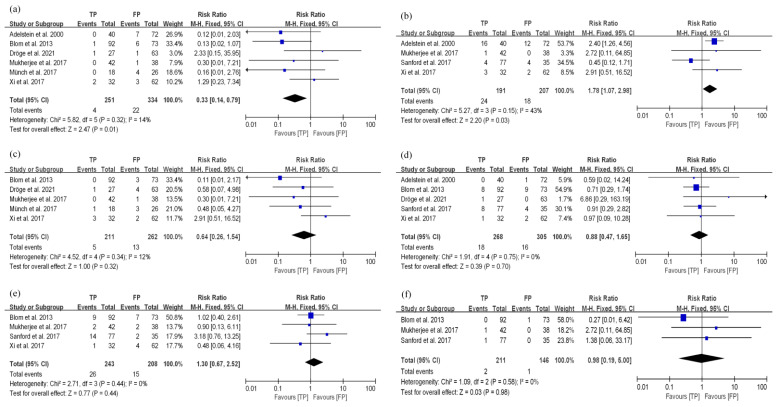
Related toxicity of TP and FP in EC patients treated with nCRT. (**a**) thrombocytopenia; (**b**) febrile neutropenia; (**c**) anemia; (**d**) nausea/vomiting; (**e**) esophagitis; (**f**) diarrhea.

For the relationship between the toxicity (grade ≥ 3) and different chemotherapy regimens in EC patients undergoing nCRT, the summary results showed that incidences of thrombocytopenia in EC patients treated with FP regimens were higher than in those undergoing TP regimens (RR: 0.33, 95% CI: 0.14–0.79, *p* = 0.01, Figure 7a). We also found that the TP regimen caused more febrile neutropenia than FP regimen (RR: 1.78, 95% CI: 1.07–2.98, *p* = 0.03, Figure 7b). However, there were no significant differences in the incidence of anemia (RR: 0.64, 95% CI: 0.26–1.54, *p* = 0.32, Figure 7c), nausea/vomiting (RR: 0.88, 95% CI: 0.47–1.65, *p* = 0.70, Figure 7d), esophagitis (RR: 1.30, 95% CI: 0.67–2.52, *p* = 0.44, Figure 7e) and diarrhea (RR: 0.98, 95% CI: 0.19–5.00, *p* = 0.98, Figure 7f) between these two groups. All the above results had no heterogeneity (Figure 7).

## 4. Discussion

EC has always been one of the most common malignant tumors worldwide [2]. Numerous measures have aimed to improve the EC prognosis of multimodal treatment, including improved surgical procedures, precise radiotherapy technologies, and the combined application of antitumor drugs [1]. FP is a recognized first-line chemotherapy regimen for EC [5,52]. With the rapid development of its clinical practice and application, the TP regimen has also proved to be effective in the treatment of EC [53]. According to the National Comprehensive Cancer Network Guideline 2022, radical chemoradiotherapy is the first-line treatment for unresectable, locally advanced EC, and nCRT has sufficient medical evidence for the treatment of resectable, locally advanced EC; this is also recommended as a routine treatment [54]. The preferred regimens include fluorouracil plus oxaliplatin, fluorouracil plus cisplatin, and paclitaxel plus carboplatin [54]. Paclitaxel plus carboplatin and fluorouracil plus oxaliplatin are the most recommended preoperative chemotherapy regimens for localized thoracic esophageal or esophagogastric junction adenocarcinomas [54]. To date, there has been no consensus on the strengths and weaknesses of TP and FP chemotherapy regimens in chemoradiotherapy for EC. The present study examined the differences in the therapeutic efficacy and related toxicity of TP and FP in EC patients, and it found that the prognosis and therapeutic response of EC patients undergoing TP–dCRT treatment were superior to those of patients treated with FP–dCRT treatment. However, these two regimens had a similar survival time in EC patients undergoing nCRT. Furthermore, our review found that TP and FP regimens differed significantly in the aspect of myelosuppression.

By summarizing, we divided the included articles into dCRT groups and nCRT groups. We found that EC patients undergoing dCRT benefited more from the TP regimen than the FP regimen, and patients who received the TP regimen had significantly longer survival time and better ORRs. A meta-analysis of 31 studies illustrated that taxane-based treatment produced better clinical responses and outcomes than FP therapy in EC patients receiving dCRT [16]. Li et al. [55] investigated 59 EC patients receiving dCRT and found that cisplatin plus paclitaxel regimens had better ORR and longer survival than cisplatin plus 5-FU regimens. Zhao et al. [28] evaluated the efficacy and safety of two chemoradiotherapy regimens (5-FU plus cisplatin and docetaxel plus cisplatin) in patients with unresectable, locally advanced ESCC. They discovered that the ORR and OS of the TP regimen were better than those of the FP regimen. These conclusions are consistent with our study’s deduction that taxane-based chemoradiotherapy seems to have better clinical benefits than a fluorouracil-based regimen in EC patients receiving dCRT. Paclitaxel produces cytotoxic activity against EC and can interfere with microtubule depolymerization and cell division, which are moderate radiosensitizers for some human tumor cells [10]. Previous studies have shown that taxanes could enhance the response to radiation by inducing mitotic arrest and apoptosis in mouse tumor cells [10,11]. The sensitization of radiotherapy might be the reason why taxanes are superior to fluorouracil in EC patients undergoing dCRT. However, it should be noted that most of the dCRT studies in this review are from Asia, and the proportion of ESCC participants is more than 90%. Whether the TP–dCRT regimen has a therapeutic advantage in EAC needs to be further studied.

Our findings demonstrate that TP and FP regimens produced similar prognoses in EC patients undergoing nCRT. Meanwhile, the pCR of the FP group was better than that of the TP group, and the R0 resection rate of the TP group was superior to that of the FP group. Similar to our results, Dröge et al. [34] found that there was no significant difference between the OS and PFS of the TP group and the FP group, and the pCR of the FP group was better than that of the TP group in patients receiving nCRT. In previous meta-analyses, taxane-based treatment and FP therapy showed similar OS, PFS, pCR, and R0 resection rates in EC patients receiving nCRT, which is not completely consistent with our results [16]. Compared with the chemotherapy regimens in the above article (including paclitaxel plus fluorouracil, paclitaxel plus fluorouracil plus platinum, and docetaxel single drug), our study refined the chemotherapy regimen into paclitaxel plus platinum or fluorouracil plus platinum, which reduced the heterogeneity caused by the chemotherapy regimens. In addition, the number of articles included in the present study is also increased. More importantly, this study improved the robustness of the results to a certain extent through sensitivity analysis. At present, there are multiple studies exploring the modes of neoadjuvant therapy, including neoadjuvant chemotherapy combined with immunotherapy and nCRT combined with immunotherapy, and some have achieved excellent results. We look forward to updated research data, which will continue to improve the existing treatment modes [56,57].

Our results demonstrated that the incidence of myelosuppression (leucopenia and thrombocytopenia) in the TP regimen was higher than that of the FP regimen in EC patients undergoing dCRT, which was consistent with the data of Zhu et al. [29]. We further compared EC patients receiving nCRT with the conclusion that FP and TP groups correspond to higher incidences of thrombocytopenia and febrile leukopenia, respectively. Blom et al. [43] also reported similar results referring to the higher thrombocytopenia rate in FP–nCRT. Several other studies reported that incidences of febrile neutropenia in TP regimens were higher than in FP regimens in EC patients receiving nCRT [37,38,45]. All these studies also proved the rationality of our investigation. Interestingly, the incidence of thrombocytopenia in different chemotherapy regimens obtained entirely different results for dCRT and nCRT. The reason for this situation might be related to the differences in drug type and dose of the chemotherapy regimens. In the nCRT group, the TP regimen was mostly paclitaxel plus carboplatin, and the FP regimen was mostly 5-FU plus cisplatin. Cisplatin is generally greater in toxicity than carboplatin and is more likely to cause renal damage, which may influence the production of thrombopoietin and reduce platelet production [25,58]. Meanwhile, among the eight included nCRT studies, the FP group in five studies had a higher fluorouracil dose, and the FP group in two studies had a greater radiation dose. These factors might have caused higher incidences of thrombocytopenia in FP regimens than in TP regimens in the nCRT group. However, the TP regimen was more likely to give rise to myelosuppression in the dCRT group. Most of the dCRT participants were elderly patients who had an increased risk of myelosuppressive-associated complications from chemotherapy. Studies have shown a decrease in the function of the bone marrow with age. Meanwhile, Huang et al. [20] demonstrated increased hematologic toxicity with taxane-based regimens in elderly EC patients treated with dCRT. In addition, the radiotherapy dose in the dCRT group was slightly higher than in the nCRT group, and a larger radiotherapy dose could also aggravate the toxicity [59]. These might be the reasons for these different results; more in-depth studies are needed in the future.

There are also some limitations in this study. Firstly, since some articles did not provide the relevant survival data directly, Eagauge Digitizer software was used to extract the data from the survival curve and calculate the results indirectly. Secondly, most of our analyzed data came from retrospective cohort studies with inherent limitations and some inevitable selection bias. Thirdly, the review was not registered, but the meta-analysis was carried out in strict accordance with the PRISMA statement. Fourthly, because of the difficulties in screening participants, this review did not conduct a subgroup analysis according to pathological types. In addition, the influence of confounding factors from some small-size studies cannot be excluded. According to the above limitations, the multicenter, high quality, and large sample size studies need to be further discussed.

## 5. Conclusions

This study indicated that taxanes combined with platinum could produce superior clinical responses and survival benefits when compared with fluorouracil combined with platinum in EC patients treated with dCRT. Meanwhile, the two treatment regimens have equivalent survival benefits for EC patients undergoing nCRT. These findings might provide guidance for clinicians to choose appropriate treatment regimens for patients with esophageal cancer.

## Figures and Tables

**Table 1:** Characteristics of 31 selected studies included in the systematic review and meta-analysis.

Authors	Year	Geographical Area	Research Type	Pathological Type (No.)	Treatment Strategy	Chemotherapy Regimen	No. of Patients (TP/FP)
Hsu et al. [18]	2008	Taiwan, China	RCS	ESCC (127)	dCRT/nCRT	PTX + DDP vs. 5-FU + DDP	57/70
Bai et al. [19]	2013	China	RCT	ESCC (71)	CCRT	DTX + DDP vs. 5-FU + DDP	35/36
Huang et al. [20]	2020	China	RCS	ESCC (46)	CCRT	PTX/DTX + DDP/CBP vs. 5-FU + DDP	22/24
Hu et al. [21]	2016	China	RCS	ESCC (202)	dCRT	PTX + DDP vs. 5-FU + DDP	105/97
Münch et al. [22]	2018	Germany	RCS	ESCC (41)	dCRT	PTX + CBP vs. 5-FU + DDP	18/23
Qu et al. [23]	2017	Canada	RCS	ESCC/EAC (26/47)	dCRT	PTX + CBP vs. 5-FU + DDP/CBP	26/47
Sun et al. [24]	2016	China	RCS	ESCC (179)	dCRT	PTX/DTX + DDP/CBP vs. 5-FU/Tegafur/FT207 + DDP/NDP	83/96
Honing et al. [25]	2013	Multicenter	RCS	ESCC/EAC (51/51)	dCRT	PTX + CBP vs. 5-FU + DDP	55/47
Fang et al. [26]	2017	China	RCS	ESCC (82)	CCRT	PTX + DDP vs. S-1 + DDP	41/41
Yang et al. [27]	2015	China	RCT	ESCC (68)	CCRT	PTX + LBP vs. 5-FU + DDP	34/34
Zhao et al. [28]	2012	China	RCT	ESCC (90)	CCRT	DTX + DDP vs. 5-FU + DDP	45/45
Zhu et al. [29]	2017	China	RCT	ESCC (86)	CCRT	DTX + DDP vs. 5-FU + DDP	45/41
Zhang et al. [30]	2016	China	RCS	ESCC (204)	dCRT	DTX + DDP vs. 5-FU + DDP	102/102
Su et al. [31]	2021	Taiwan, China	PCS	ESCC/EAC (133/3)	CCRT/nCRT	PTX + CBP vs. 5-FU + DDP	87/49
Jiang et al. [32]	2020	Canada	RCS	ESCC/EAC (34/59)	dCRT/nCRT	PTX + CBP vs. 5-FU + DDP	40/53
Hsieh et al. [33]	2021	Taiwan, China	RCS	ESCC (229)	CCRT/nCRT	PTX + CBP vs. 5-FU + DDP	83/146
Dröge et al. [34]	2021	Germany	RCS	ESCC (90)	nCRT	PTX + CBP vs. 5-FU + DDP	27/63
Wong et al. [35]	2020	Hong Kong, China	RCS	ESCC (200)	nCRT	PTX + CBP vs. 5-FU + DDP	100/100
Bajwa et al. [36]	2018	India	RCS	ESCC/EAC (38/12)	nCRT	PTX + CBP vs. 5-FU/Cape + DDP	30/20
Xi et al. [37]	2017	China	RCS	ESCC (94)	nCRT	DTX + DDP vs. 5-FU + DDP	32/62
Sanford et al. [38]	2017	America	RCS	ESCC/EAC (18/94)	nCRT	PTX + CBP vs. 5-FU + DDP	77/35
Jipping et al. [39]	2017	Netherlands	RCS	ESCC/EAC (22/104)	nCRT	PTX + CBP vs. 5-FU + DDP	63/63
Haisley et al. [40]	2017	Australia	RCS	ESCC/EAC (20/122)	nCRT	PTX + CBP vs. 5-FU + DDP	87/55
Duff et al. [41]	2017	America	RCS	ESCC/EAC (3/35)	nCRT	PTX + CBP vs. 5-FU + DDP	14/24
Boggs et al. [42]	2014	America	RCS	ESCC/EAC (44/115)	nCRT	PTX + DDP/CBP vs. 5-FU + DDP	30/129
Blom et al. [43]	2013	Netherlands	RCS	ESCC/EAC/other (39/124/2)	nCRT	PTX + CBP vs. 5-FU + DDP	92/73
Orditura et al. [44]	2011	Italy	RCS	ESCC/EAC (54/18)	nCRT	PTX + DDP vs. 5-FU + DDP	33/39
Adelstein et al. [45]	2000	America	RCS	ESCC/EAC/other (29/70/3)	nCRT	PTX + DDP vs. 5-FU + DDP	40/62
Münch et al. [46]	2017	Germany	RCS	ESCC (44)	nCRT	PTX + CBP vs. 5-FU + DDP	18/26
Tamtai et al. [47]	2017	Thailand	RCS	ESCC/EAC/other (113/6/5)	CCRT/nCRT	PTX + CBP vs. 5-FU + Platinum	60/64
Mukherjee et al. [48]	2017	Multicenter	RCT	EAC (85)	nCRT	PTX + CBP vs. Cape + OXA	43/42

Abbreviations: 5-FU, fluorouracil; Cape, capecitabine; CBP, carboplatin; CCRT, concurrent chemoradiotherapy; dCRT, definitive chemoradiotherapy; DDP, cisplatin; DTX, docetaxel; EAC, esophageal adenocarcinoma; ESCC, esophageal squamous cell carcinoma; LBP, lobaplatin; nCRT, neoadjuvant chemoradiotherapy; NDP, nedaplatin; OXA, oxaliplatin; PTX, paclitaxel; PCS, prospective cohort study; RCS, retrospective cohort study; RCT, randomized controlled trial.

**Table 2:** Symmetry test of funnel plots.

	3-Year OS of dCRT	3-Year PFS of dCRT	3-Year OS of nCRT	3-Year PFS of nCRT	pCR	R0 Resection
*Begg’s* test	0.837	0.858	0.304	1.000	0.300	0.174
*Egger’s* test	0.727	0.410	0.110	0.788	0.342	0.210

Abbreviations: OS, overall survival; pCR, pathologic complete response; PFS, progression free survival.

## Supplementary Materials

The following supporting information can be downloaded at: https://www.mdpi.com/article/10.3390/curroncol29090519/s1, Figure S1. Sensitivity analysis. (a) 3-year PFS in dCRT; (b) 3-year OS in nCRT; (c) 3-year PFS in nCRT; (d) thrombocytopenia in dCRT; (e) nausea/vomiting in dCRT. Table S1. Survival rate of 31 selected studies included in the systematic review and meta-analysis.
