# Supplementary material for: Comparison of Efficacy and Safety of Taxanes Plus Platinum and Fluorouracil Plus Platinum in the First-Line Treatment of Esophageal Cancer: A Systematic Review and Meta-Analysis
Source: Curr Oncol. 2022 Sep 16;29(9):6610–27. doi: 10.3390/curroncol29090519 (PMC9497974; doi:10.3390/curroncol29090519)
Supplement: Supplementary file 1 [file curroncol-29-00519-s001.zip › curroncol-1844672-supplementary.pdf]

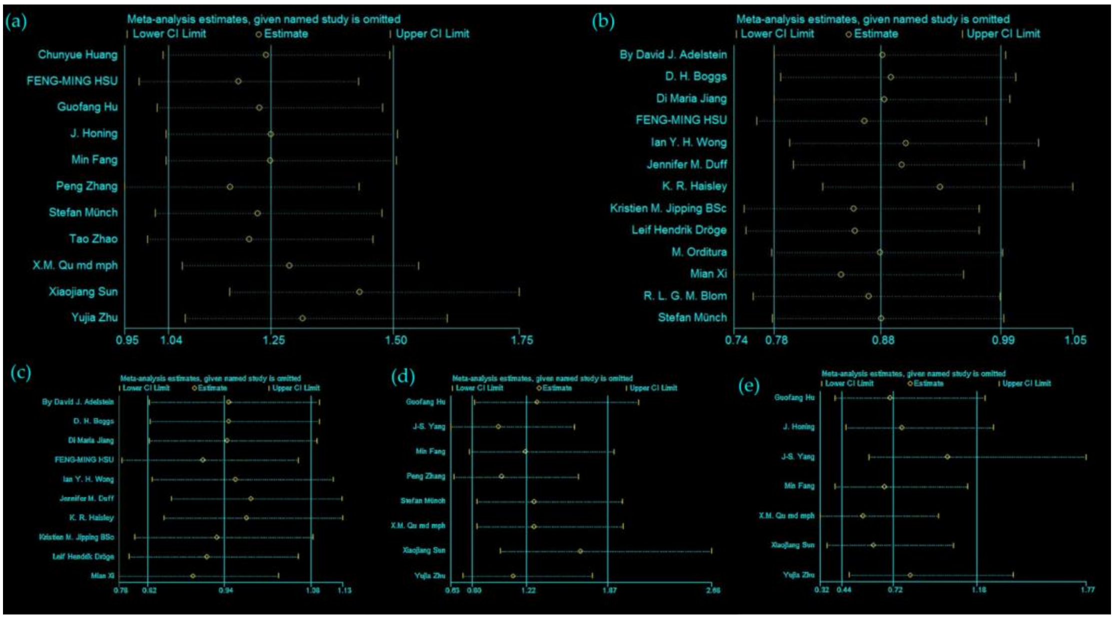

Figure S1. Sensitivity analysis. (a) 3-year PFS in dCRT ; (b) 3-year OS in nCRT ; (c) 3-year PFS in nCRT ; (d) thrombocytopenia in dCRT ; (e) nausea/vomiting in dCRT.

Table S1. Survival rate of 31 selected studies included in the systematic review and meta-analysis.

Authors	Treatment strategy	3-year OS rates (TP/FP)	*P*	3-year PFS rates (TP/FP)	*P*
Hsu et al. [18]	dCRT/nCRT	dCRT：55%/31%<br>nCRT：46%/41%*	0.12<br>--	dCRT：67%/19%*<br>nCRT：77%/66%*	--<br>--
Bai et al. [19]	CCRT	49%/42%*	--	--	--
Huang et al. [20]	CCRT	45%/42%*	--	32%/21%*	--
Hu et al. [21]	dCRT	48%/32%*	--	13%/8%*	--
Münch et al. [22]	dCRT	56%/39%*	--	56%/30%*	--
Qu et al. [23]	dCRT	15%/40%	--	19%/28%*	--
Sun et al. [24]	dCRT	39%/34%*	--	29%/41%*	--
Honing et al. [25]	dCRT	29%/21%*	--	24%/19%*	--
Fang et al. [26]	CCRT	32.4%/32.1%	0.796	24.9%/18.9%	0.63
Yang et al. [27]	CCRT	--	--	--	--
Zhao et al. [28]	CCRT	44%/24%*	--	38%/20%*	--
Zhu et al. [29]	CCRT	60%/64%*	--	51%/56%*	--
Zhang et al. [30]	dCRT	39%/25%*	--	40%/25%*	--
Su et al. [31]	CCRT/nCRT	--	--	--	--
Jiang et al. [32]	dCRT/nCRT	dCRT：12%/63%<br>nCRT：50%/64%	0.001<br>0.46	dCRT：0/31%<br>nCRT：37%/44%	0.004<br>0.67
Hsieh et al. [33]	CCRT/nCRT	--	--	--	--
Dröge et al. [34]	nCRT	67%/56%*	--	63%/52%*	--
Wong et al. [35]	nCRT	36%/48%*	--	47%/56%*	--
Bajwa et al. [36]	nCRT	--	--	--	--
Xi et al. [37]	nCRT	64.9%/46%	0.039	45.9%/24.2%	0.044
Sanford et al. [38]	nCRT	--	--	--	--
Jipping et al. [39]	nCRT	54%/49%*	--	56%/56%*	--
Haisley et al. [40]	nCRT	24%/52%	--	31%/44%	--
Duff et al. [41]	nCRT	7%/46%*	--	0/54%*	--
Boggs et al. [42]	nCRT	36%/48%	--	33%/39%	--
Blom et al. [43]	nCRT	57%/61%	0.725	--	--
Orditura et al. [44]	nCRT	35%/40%	--	--	--
Adelstein et al. [45]	nCRT	30%/36%	0.45	36%/41%	--
Münch et al. [46]	nCRT	50%/58%	--	--	--
Tamtai et al. [47]	CCRT/nCRT	--	--	--	--
Mukherjee et al. [48]	nCRT	--	--	--	--

* Data extracted by Eagauge Digitizer software.

Abbreviations: CCRT, concurrent chemoradiotherapy; dCRT, defin-itive chemoradiotherapy; nCRT, neoadjuvant chemoradiotherapy; OS, overall survival; PFS, progression-free survival.
